# Enhancements in Radiological Detection of Metastatic Lymph Nodes Utilizing AI-Assisted Ultrasound Imaging Data and the Lymph Node Reporting and Data System Scale

**DOI:** 10.3390/cancers16081564

**Published:** 2024-04-19

**Authors:** Cezary Chudobiński, Bartosz Świderski, Izabella Antoniuk, Jarosław Kurek

**Affiliations:** 1 Copernicus Regional Multi-Specialty Oncology and Trauma Centre, 93-513 Lódź, Poland; cezary.chudobinski@wp.pl; 2 Department of Artificial Intelligence, Institute of Information Technology, Warsaw University of Life Sciences, 02-776 Warsaw, Poland; bartosz_swiderski@sggw.edu.pl (B.Ś.); izabella_antoniuk@sggw.edu.pl (I.A.)

**Keywords:** LN-RADS, lymph nodes, ultrasound, metastasis diagnosis, artificial intelligence

## Abstract

**Simple Summary:**

A novel approach for automatic detection of neoplastic lesions in lymph nodes is presented, which incorporates machine learning methods and the new LN-RADS scale. The presented solution incorporates different network structures with diverse datasets to improve the overall effectiveness. Final findings demonstrate that incorporating the LN-RADS scale labels improved the overall diagnosis, especially when compared with current, standard practices. The presented solution is meant as an aid in the diagnosis process.

**Abstract:**

The paper presents a novel approach for the automatic detection of neoplastic lesions in lymph nodes (LNs). It leverages the latest advances in machine learning (ML) with the LN Reporting and Data System (LN-RADS) scale. By integrating diverse datasets and network structures, the research investigates the effectiveness of ML algorithms in improving diagnostic accuracy and automation potential. Both Multinominal Logistic Regression (MLR)-integrated and fully connected neuron layers are included in the analysis. The methods were trained using three variants of combinations of histopathological data and LN-RADS scale labels to assess their utility. The findings demonstrate that the LN-RADS scale improves prediction accuracy. MLR integration is shown to achieve higher accuracy, while the fully connected neuron approach excels in AUC performance. All of the above suggests a possibility for significant improvement in the early detection and prognosis of cancer using AI techniques. The study underlines the importance of further exploration into combined datasets and network architectures, which could potentially lead to even greater improvements in the diagnostic process.

## 1. Introduction

The lymph nodes (LNs) are the crucial part of oncological staging. High accuracy of metastatic LN detection is an important factor for treatment outcomes. In the TNM (Tumor Nodes Metastasis) system, metastases to regional lymph nodes are treated as nodal involvement. Changing from the N0 to N1 or higher category significantly worsens the prognosis. Metastases to distant nodes are treated in the TNM system as organ metastases—the M0 category changes to M1 or higher, which lowers the chances of recovery even more.

The involvement of lymph nodes in head and neck squamosus cell carcinoma is the most important prognostic factor [1]. According to Som, the presence of a single ipsilateral or contralateral metastatic node reduces survival by 50% and bilateral disease by a further 50% [2]. In the case of prostate cancer, patients who had one or two positive lymph nodes had a clinical recurrence-free survival of 70% and 73% at 10 years, respectively, vs. 49% in those who had five or more involved lymph nodes (*p* = 0.0031) [3]. In the case of breast cancer, lymph nodes are of key importance in the prognosis, and regardless of the existing treatment options, a precise determination of the number of lymph nodes affects the success of treatment [4,5]. The author of [6] argues that it is very important to precisely indicate nodes with metastases and confirm them with biopsy. This is an expression of the use of precision medicine individually tailored to each patient and not relying on general statistical calculations and routine removal of lymph nodes. According to Rebekah R, the most powerful predictor of survival in melanoma malignum was the number of positive lymph nodes. Stratification by this factor alone reveals a wide range of estimated 5-year survival rates, from 53% for patients with one positive node to 25% for patients with more than four positive nodes. This observation has been so consistent that the number of positive lymph nodes replaced gross dimension in the N classification of the recently revised American Joint Committee on Cancer (AJCC) staging system, 23, with N1, N2, and N3 designating one, two or three, and four or more nodes, respectively [7].

Knowing the prognostic importance of nodal involvement, SLND sentinel node removal is used in many situations based on the stage of the primary tumor. In some cases, the node does not contain metastases and the procedure is mutilating for the patient. Some researchers advocate a critical approach to preventive removal of nodes and recommend chemotherapy [8]. However, chemotherapy is also not indifferent to the body and should not be used in people who do not have metastases in the nodes. Therefore, it is important to detect small metastases in the nodes and look for methods that will allow for a better determination of the stage of advancement. This will allow the use of lymphadenectomy, chemotherapy, or radiotherapy only in cases of the actual presence of metastases in the nodes. Radical tumor removal alone does not guarantee the success of the therapy. Leaving metastatic nodes will often result in recurrence and development of cancer. Lymph nodes, as filters capturing cancer cells, should be treated, especially at the stage of initial diagnosis and staging (determining the stage of advancement), but also throughout the course of treatment and after its completion as they may be the site of cancer recurrence.

The accurate detection and characterization of metastatic involvement in LNs is a critical factor in the staging and treatment planning for various malignancies. Knowing this, researchers have been searching for appropriate diagnostic methods. Despite its significance, there is a lack of consensus regarding the criteria for assessing LN metastasis, leading to variability in diagnostic approaches and outcomes. Traditional methods have heavily relied on size-based parameters, such as the short-axis diameter (SAD), long-axis diameter (LAD), volume, or certain chosen structural features: shape, internal structure, borders. Finally, each method obtains different cut-off values with different specificity, sensitivity, and accuracy. However, emerging evidence suggests that size alone may not be a definitive indicator of the nature of LN changes, and a more comprehensive evaluation of morphological and structural characteristics is warranted. In some papers, the authors concentrate prediction on the assessment of LNs only, while in others, they underline the strength of additional clinical factors—among them, histological tumor grade (e.g., Gleason score), value of Ki67, or level of tumor markers [9,10,11,12].

Contemporary radiological practices encompass a wide range of criteria for LN assessment, including diverse imaging modalities and techniques. While some studies focus solely on ultrasound B-mode imaging, others incorporate additional parameters such as color/power Doppler, elastography, or advanced MRI sequences like diffusion-weighted imaging (DWI) and apparent diffusion coefficient (ADC) maps. Furthermore, the advent of novel molecular imaging techniques, such as ultra-small superparamagnetic iron oxide (USPIO) nanoparticles, has opened new avenues for improving LN evaluation [13,14].

Jager and Barentsz et al. utilize computer tomography (CT) and magnetic resonance imaging (MRI), where the determination of metastatic LNs is performed largely by size. Thresholds of 10 mm for the short axis of oval nodes and 8 mm for round nodes are generally used as indicators of likely metastatic disease [15]. However, Davis claims that more than half of LNs involved with metastatic prostate cancer may be less than 10 mm [16]. Moreover, non-metastatic nodes may be enlarged due to reactive hyperplasia. Given the lack of sensitivity of both CT and MRI based on size criteria alone, new techniques of MR lymphography (MRL) have been developed as well as molecular imaging techniques. Louise Benoit et al. analyzed the usefulness of the 10 mm cut-off value for prediction of LNs metastases in advanced ovarian cancer. The presence of enlarged LNs on CT turned out to be a weak indicator of LN involvement in patients undergoing Neoadjuvant Chemotherapy (NACT). However, it could be used to assess the overall prognosis [17]. The study by Sohn et al. [18] focuses on evaluating LN metastasis from thyroid cancer using ultrasound. The study does not use SAD criteria and shows that the most accurate ultrasound criteria for differentiating metastatic from benign LNs are subjecive rather than measurable: loss of fatty hilum, cystic change, calcification, hyperechogenicity (higher echogenicity than the surrounding muscles), and round shape.

A.T. Ahuja et al. [19] offer a comprehensive review of the sonographic features for assessing metastatic and lymphomatous cervical LNs—various greyscale and Doppler sonographic features such as nodal size, shape, echogenicity, and vascularity to differentiate malignant and benign nodes. It is noted that lymphomatous nodes tend to be enlarged with a minimum transverse diameter of 10 mm or larger, but nodal size alone is not an accurate criterion for differentiating lymphomatous nodes from normal or other pathological LNs.

Alvarez performed a MEDLINE search (keywords, “sonography” OR “ultrasound” AND “axillary”) and a manual search of the references of relevant studies and reviews of preoperative diagnosis on sonography of possible axillary metastases. Sixteen articles were selected. In sonography of axillae without palpable nodes, and using LN size as the criterion for positivity, sensitivity varied between 48.8% (95% confidence interval, 39.6–58%) and 87.1% (76.1–94.3%) and specificity between 55.6% (44.7–66.3%) and 97.3% (86.1–99.9%). When LN morphology was used as the criterion for positivity, sensitivity ranged from 26.4% (15.3–40.3%) to 75.9% (56.4–89.7%) and specificity from 88.4% (82.1–93.1%) to 98.1% (90.1–99.9%). The results are different if axillae with palpable nodes are included. The sonographically guided biopsy shows a sensitivity that varies between 30.6% (22.5–39.6%) and 62.9% (49.7–74.8%) and a specificity of 100% (94.8–100%). Many of the summary results obtained after meta-analysis show a heterogeneity that disappears, on occasion, when excluding the studies that use a double gold standard [20].

Despite these advancements, the lack of a standardized and universally accepted system for LN assessment has hindered effective communication and decision-making among clinicians and radiologists. The LN-RADS, a multiparametric structural approach, aims to address this gap by providing a simple and intuitive scale for characterizing LNs. This system has been validated in a study analyzing ultrasound images of 512 LNs, demonstrating its value in differentiating metastatic from non-metastatic LNs and achieving good inter-reader agreement. The structural assessment allows the detection of small macrometastases (even 3 mm) significantly earlier than when referring to the the SAD size threshold (10 mm) often used in various scientific articles [17,21,22,23,24,25,26,27,28,29,30,31].

The role of artificial intelligence (AI) in diagnostic imaging has garnered significant attention due to its potential to enhance accuracy and efficiency. By leveraging machine learning (ML) techniques and integrating diverse datasets, AI-assisted methods can potentially improve the early detection and prognostic evaluation of neoplastic lesions in LNs. This study investigates the utility of the LN-RADS scale in conjunction with AI algorithms, aiming to develop an automated system for the accurate identification of metastatic LN involvement.

We notice that, in the context of AI learning, LN-RADS with a six-point scale (1, 2, 3, 4a, 4b, 5) can perform better than the zero–one system of histopatological diagnoses (0—benign, 1—malignant). On the one side, final histopathological diagnosis is defined binarily (0 or 1), and on the other side, diversity of appearance of the LNs is infinite. Therefore, we tried to use the LN-RADS scale to segregate similar patterns and create groups of LNs with a similar percentage of malignancy risk, giving AI several intermediate states instead of two extreme values of histopathological results. In the case of learning based on histopathological binary diagnoses, AI recognizes nodes with extremely malignant or benign patterns well but has difficulty recognizing images with intermediate features. The LN-RADS scale allows for more effective training and differentiation of nodes with intermediate features and, as a result, increases the effectiveness of the method.

Nowadays, we perceive the role of AI as a supporting tool rather than replacing the radiologists. It can help identify subtle patterns and changes that may not be immediately apparent to the human eye, thus enhancing diagnostic accuracy. In doing so, AI-driven CAD systems hold the promise of changing LN evaluation, supporting personalized patient care, and ultimately improving oncological outcomes.

### Stages of Metastatic Progression

**Figure 1:**
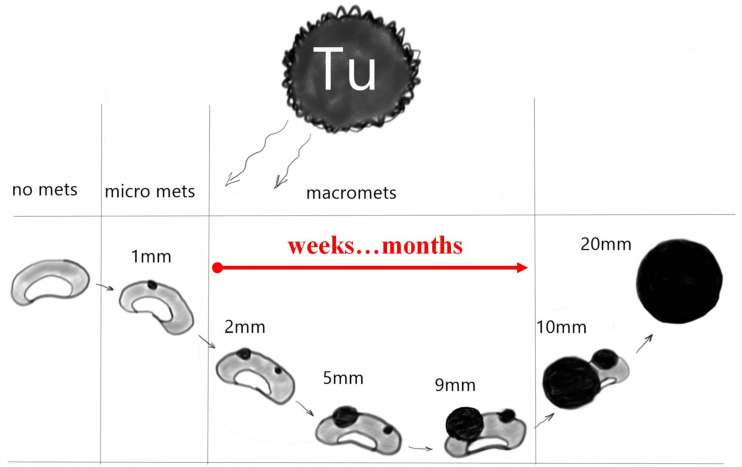
Schematic representation of metastatic progression in LNs over time. Tu—tumor. mets—metastases. The goal is the detection of small metastases.

The progression of metastatic lesions in the LNs is a critical factor in the staging and treatment of cancer. Figure 1 illustrates the typical progression from no metastases to micro-metastases and ultimately to macro-metastases. The process evolves from no visible metastases to micro-level involvement and finally to significant macro-level metastases. The timeline indicates that these changes can occur over a period of weeks to months, highlighting the rapidity with which cancer can progress.

The successive stages are defined as follows:

- No Metastasis: Initially, the LNs are cancer-free.
- Micrometastases: This is characterized by the presence of tiny nests of cancer cells within the LN, starting at a size as small as 0.2 mm and progressing to 2 mm. These represent early-stage metastasis and might be clinically undetectable.
- Macrometastases: For the nodes between sizes 2 mm and 9 mm, the metastasis is classified as macrometastasis. The progression to a 20 mm lesion represents a significant growth of the cancer within the LN, possibly indicating advanced disease.

The stage of cancer, including the stage of LN involvement, affects the type of treatment and the final outcomes. The more advanced cases require aggressive treatment and have a worse prognosis. Therefore, precise and early detection of small cancer lesions in LNs is critical in this process.

There are few factors in the AI training process that can influence the overall accuracy, especially when deep learning or Convolutional Neural Networks (CNNs) are concerned [32,33,34]. The first of them would be generalization and whenever the resulting model can analyze the data instead of just memorizing presented examples. This ability is one of the main indicators showing if the network is effective [35,36]. Additionally, since in many cases the testing data can be considered as training data with added noise, in order to improve the overall network generalization, the process of noise reduction should also be considered while preparing the solution. Depending on the considered problem, different methods might be applied, like the evaluation of view stability [37] or group-based restoration methods [38].

This issue only grows in the case of medical image analysis. Since such problems are complex and require the precise recognition of chosen elements, large neural structures will usually be needed. At the same time, since the process of marking samples is lengthy in most cases, the number of available examples can be limited. This can pose a huge problem for networks, especially when the generalization heavily depends not only on the overall network structure but also on the population of available learning data [39,40].

The problem of generalization in neural networks is a widely discussed one, with different methods developed in order to improve it [41,42,43,44,45]. For example, in [46], a new image classification task was introduced in order to evaluate the CNN and Capsule Network’s generalization ability. In case of Top-2 classification, models were trained on single-label image samples, while in the test stage, some samples were randomly concatenated from two differently denoted ones. The goal was set to predict the Top-2 labels on the created images, while the evaluation provided some insight into each network’s generalization capabilities, with the Capsule Network greatly exceeding the standard CNN results. In [47], norm-based control, sharpness, and robustness are considered as a generalization measure, while the authors of [48] explore the reasons behind good generalization in large neural networks.

One interesting approach to increasing the generalization capability is using a network ensemble instead of a single model. In such cases, each network works independently, while the final result is achieved by some type of voting between chosen classifiers [41]. The differences between individual models can include using randomly chosen subsets of training data and diversifying the network structure or dropout ratio. Another approach builds CNNs with a random choice of layers and activation function (either ReLU or softmax). The performed experiments have shown significant improvement in the accuracy, sensitivity, specificity, precision, F1, and area under the ROC curve scores achieved [49].

The detection and accurate characterization of LNs in the context of cancer diagnosis remain one of the pivotal challenges in medical imaging that has profound implications on treatment pathways and patient prognoses. Traditional beliefs have held the short-axis diameter (10 mm SAD threshold [17,21,22,23,24,25,26,27,28,29,30,31]) of LNs as a critical parameter. However, recent studies [28,50,51] have cast new light on this aspect, suggesting that size may not be the definitive criterion for the nature of LN changes.

This paper aims to investigate the utility of the Lymph Node Reporting and Data System (LN-RADS) in the diagnostic imaging of LNs for the automatic detection of neoplastic changes. The identification of malignant transformations in LNs is a key issue that dictates the course of further treatment and the prognostic outlook of patients. Recent insights have pointed out that the mentioned size parameter does not conclusively determine the character of changes in an LN. A more accurate assessment, and consequently the risk of neoplastic alterations, relies on a detailed examination of the node’s appearance and internal structure.

On the other hand, the ability to detect changes early is understandably vital for the success of subsequent treatments. This interest in the early detection phase has spurred research into artificial intelligence (AI) methods in diagnostic processes. However, in supervised learning models, a natural question arises: what should be the target (element presented to the network as the correct, desired response)? While histopathological outcomes (confirming or ruling out malignancy) seem to be the ideal candidate since the goal is to predict “cancer”, this approach does not always yield the best results. Binary histopathological data (0 or 1) are good at teaching extremely cancerous or healthy LNs but not those presenting intermediate forms.

To address the problem resulting from histopatological samples, the novel LN-RADS scale is used to describe them. In the presented research, a total of four methods are evaluated and compared. The first two approaches use the original denotations from the histopathological data, marking the samples as tumorous or not for the learning process. The second set uses the LN-RADS scale. The baselines for both approaches were the results achieved by using the 10 mm SAD threshold. The assumption driving the presented research was that incorporating the AI methods in the diagnostic process can significantly improve diagnostic accuracy and speed up the entire process, improving patient prognosis.

## 2. Materials and Methods

### 2.1. Materials

We conducted a retrospective, single-center study by performing ultrasound (USG) on 512 superficial LNs from the years 2015 to 2021. The patient cohort consisted of 341 individuals, with 223 men and 120 women, ranging in age from 20 to 91 years, with a mean age of 59 years. Table 1 presents a detailed breakdown of the LN samples collected for this research. The table categorizes the LNs based on their anatomical location, such as axilla, neck, groin, and other unspecified areas. For each location, the table lists the number of cases observed and their respective percentages of the total sample size. This distribution is crucial for understanding the prevalence of LN involvement in different body areas, which may have implications for diagnosis and treatment strategies in oncological care. The diverse range of locations also ensures a comprehensive representation of LN characteristics, contributing to the robustness of the study’s findings.

In the initial phase of our study, a total of 512 superficial LNs were identified for potential inclusion in the dataset. However, upon closer examination, it was crucial to ensure that the ultrasound images with the highest quality and reliability will be used for analysis. To this end, a rigorous selection process was implemented to identify images that were free from artifacts or any other factors that could potentially impact the final evaluation and accuracy of the machine learning models used.

This selection process involved a detailed review of each ultrasound image, assessing factors such as image clarity, presence of artifacts, and overall suitability for inclusion in a machine learning dataset. Images that did not meet those stringent criteria for quality from an AI point of view were excluded from the dataset. These excluded images either had significant artifacts or were of a quality that could potentially introduce bias or inaccuracies into the model training and validation processes.

As a result of this meticulous screening process, the final dataset comprised 398 ultrasound images of LNs. This reduced dataset ensures a higher level of data integrity and quality from an AI point of view, which is fundamental for the development of reliable and accurate AI-driven diagnostic tools. The exclusion of 114 LNs from the original pool of 512 was thus a necessary measure to uphold the standards of our study and to ensure that the subsequent analysis and findings are based on the most reliable and high-quality data available.

In total, 398 B-mode ultrasounds were performed. All ultrasonographic observations were subsequently compared to the histopathology results obtained from 247 fine-needle biopsies (FNBs), 132 core-needle biopsies (CNBs), 18 surgical biopsies, and 1 vacuum-assisted biopsy (VAB).

The ultrasound studies were carried out by consultant radiologists experienced in oncology imaging and biopsy procedures. High-resolution probes were used within the frequency range of 8-13MHz, optimized for superficial structures. Only images of sufficient quality, as determined from the hard drives of the ultrasound scanners, were included in the study. Any examinations yielding images of poor quality or with incomplete data were rigorously excluded from further analysis.

The dataset utilized in this study comprises various samples that have been categorized based on the LN-RADS scale, which is a systematic approach used to classify LNs in the context of radiological assessments. The structure of this dataset is presented in Table 2. The table is designed to provide an intuitive understanding of the distribution and characteristics of the samples within the dataset. Each row within the table represents a unique sample, detailing its respective LN-RADS classification along with other pertinent attributes such as size, location, and the presence of any distinctive imaging features that are relevant to the LN-RADS criteria. This structured compilation of data serves as a foundational element for subsequent analysis, facilitating a comprehensive evaluation of the diagnostic implications of the LN-RADS categorizations.

Table 3 categorizes the diversity of cancer types within our dataset according to the histopathological findings. Each row represents a distinct type of cancer and enumerates the number of cases observed for that specific type. Additional columns provide details on the frequency of each cancer type, along with pertinent notes on histological characteristics and patterns observed. This table is essential for understanding the histopathological landscape of the cancers included in our study and for identifying any correlations between histopathological features and clinical outcomes.

**Figure 2:**
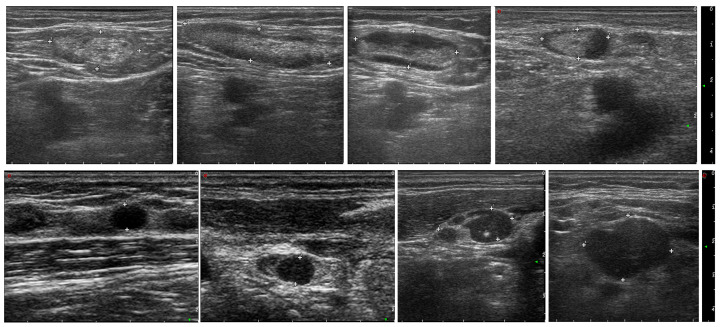
Example USG images of lymph nodes, representing benign (**upper row**) and malignant cases (**bottom row**).

Example USG images of LNs for both cancerous and non-cancerous cases are presented in Figure 2. Ultrasound imaging is a non-invasive diagnostic technique that uses high-frequency sound waves to produce images of structures within the body. In these images, the LNs are marked with plus signs (+) to outline their borders and dimensions.

The malignant LNs depicted in the bottom row show different characteristics:

- They may be larger in size.
- The shape can be more irregular or rounded rather than oval.
- The internal structure appears more heterogeneous with less clear differentiation between the cortex and hilum.
- They may show signs of infiltration to the surrounding tissues.

These images are used by healthcare professionals to aid in the diagnosis and staging of LN involvement in cancer. The evaluation of the sonographic characteristics of LNs is essential in oncology for treatment planning and prognosis.

### 2.2. The LN-RADS Scale

**Figure 3:**
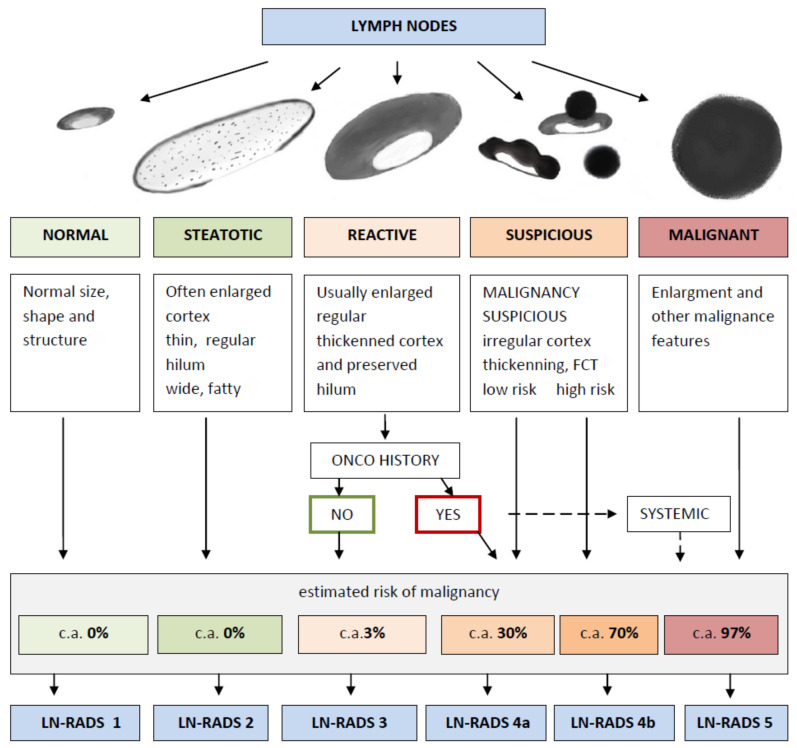
Flowchart illustrating the LN-RADS classification system.

The Lymph Node Reporting and Data System (LN-RADS) is a structured reporting protocol developed to categorize LNs based on their imaging characteristics, with the intent of standardizing diagnoses. This system is similar to the BI-RADS for breast imaging and aims to facilitate communication and decision-making among medical professionals. The scale provides a classification from LN-RADS 1 to LN-RADS 5, each corresponding to a specific set of features and management strategies. The overview of the LN-RADS classification system is shown in Figure 3.

LN-RADS Categories:

- LN-RADS 1: Normal. No enlargement (recommended max SAD up to 6–7 mm), oval shape (L/S-ratio > 2), regular cortex max thickness ≤ 3 mm, cortex echogenicity similar or higher to the background fatty tissue, smooth margins, no other changes in architecture (no calcifications, no fluid collections, no necrosis, no FCT), no pathological peripheral or chaotic vascularization
- LN-RADS 2: Steatotic. LNs enlarged in one or both axes, cortex regular, max thickness ≤ 3 mm, hilum hyperechoic (steatotic) with no size limits, no other changes in architecture (no calcifications, no fluid collections, no necrosis, no FCT), no pathological peripheral or chaotic vascularization)
- LN-RADS 3: Reactive. Probably due to inflammatory process or vaccination. Dominant feature: regular thickened cortex > 3 mm, enlargement in one or two axes, preserved oval shape (L/S-ratio ≤ 2), preserved medulla, no other changes in architecture (no calcifications, no fluid collections, no necrosis, no focal cortical thickening—FCT), cortex echogenicity similar to or moderately lower than the background fatty tissue, well-defined margins, no pathologic peripheral or chaotic vascularization, no oncological or hematological history, no laboratory oncological abnormalities
- LN-RADS 4: Suspicious for Malignancy. 4a represents low probability and 4b represents high probability of malignancy. This group is dedicated to LNs that morphologically do not match group 1, 2, 3, 5 or have additional radiological or clinical factors increasing probability of malignancy in LNs categorized as LN-RADS 3, i.e., high or increasing laboratory markers (i.e., PSA for inguinal LNs), active neoplasm in the region (i.e., breast cancer for axillary LNs), another metastatic or systemic LN in the region, clinical symptoms suggesting oncological or systemic hematological disease. The main rule of selecting LNs for group 4 is “better check than miss”. The LN-RADS 4 category is divided into two subcategories:
  – 4a: low suspicion for malignancy—size may be normal in SAD and LAD, cortex with thickening up to 4 mm, moderate irregularity, especially local. In the assumption that all 4a LNs should be verified in biopsy or PET, if it is not possible, they should be treated as suspected and malignant.
  – 4b: high suspicion of malignancy—size may be normal in SAD and LAD; cortex thickening over 4 mm and irregularity, especially FCT, or no hilum; shape more round than oval (L/S-ratio ≤ 2); hypoechogenicity to background fatty tissue, especially nearly anechoic “black hole sign”; micro-calcifications; fluid collections; necrosis; abnormal peripheral or chaotic vascularization architecture; ill-defined/blurred margins.
- LN-RADS 5: Definitely Malignant. Enlargement in SAD and one or more malignancy features: lack of hilum, hypoechogenicity to the background fatty tissue or “black hole sign”, evident cortex irregularity/FCT, shape more round than oval (L/S-ratio ≤ 2), micro-calcifications, fluid collections, necrosis, abnormal peripheral or chaotic vascularization architecture, ill-defined/blurred borders, or signs of extracapsular infiltration.

### 2.3. Methods

In order to accurately evaluate the proposed methodology, the following approaches were tested during experiments presented in this paper:

- Evaluation, using 10 mm SAD threshold—if the LN SAD is greater than 10 mm, it is assumed that the LN can be malignant;
- Training using the binary classification with the histopathological results (cancer/no cancer)—two approaches with different network structure;
- Training using the LN-RADS scale with six-point scale (1, 2, 3, 4a, 4b, 5), where the risk of malignancy increases from class ‘1’, containing mainly benign cancerous changes, to class ‘5’, consisting mainly of cancerous examples—two approaches with different network structures.

Regardless of the chosen training approach, the testing involved the binary classification with the histopathological result denoting whenever the sample contained cancerous cells. The data were divided into training/test sets. The experiments were carried out using the five-fold cross-validation, repeated 10 times, while taking into account the stratification of the target variable (similar fractions, shares of cancerous lesions in each experiment) [52]. The partial results presented further on resulted from the averaging procedure after 50 learning processes.

All performed experiments were using the RESNET-18 network as a base, with the learning process set at 100 epochs. Stochastic gradient descent (SGD) optimization was incorporated in the process, and the learning step was variable: every 10 epochs, the learning step was reduced 10 times (starting with learning rate equal to 0.1). The momentum parameter was equal to 0.1.

In order for the results to be comparable (number of parameters/network capacity), the networks trained only for the binary target variable (cancer/non-cancer) were tested in two versions, with additional neurons introduced (comparability with the LN-RADS architecture) and without this additional layer [53,54,55].

#### 2.3.1. 10 mm SAD Threshold Evaluation

The 10 mm SAD threshold provides a good practice approach [17,21,22,23,24,25,26,27,28,29,30,31] for measuring tumor burden and assessing the response to therapy in cancer clinical trials [56]. The criteria predominantly rely on changes in the size of tumors, with a particular focus on the short axis in the cross-section of the LNs, to determine the presence or absence of metastatic cancer. By convention, LNs with a short axis less than 10 mm are not considered indicative of metastatic disease.

In the context of our research, the 10 mm SAD threshold served as a comparative baseline for evaluating two other methods of tumor assessment. Unlike the other methods under study, the 10 mm SAD threshold is non-computational; it is a binary classification where the sample is directly labeled as cancerous or non-cancerous based on the short-axis measurement of the LN’s cross-section.

**Figure 4:**
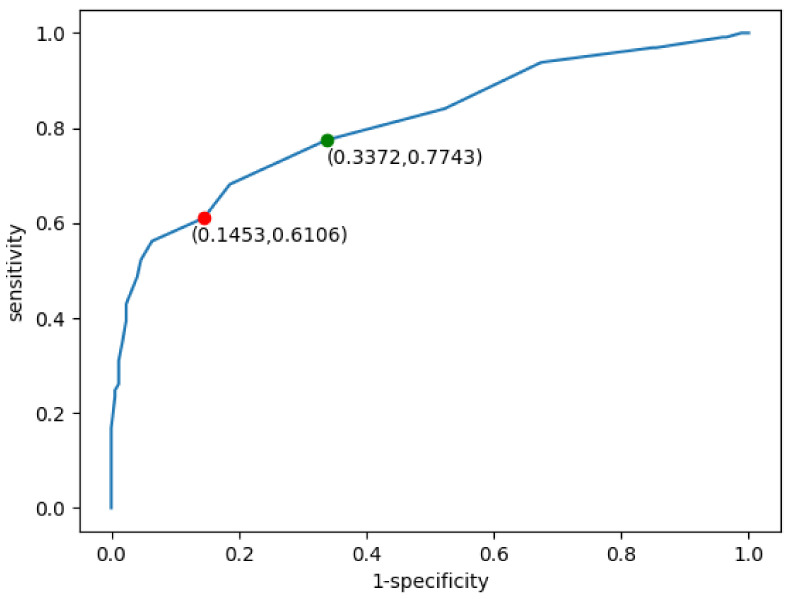
ROC graph for the sample classification with the 10 mm SAD threshold (red dot) and 8 mm optimal threshold (green dot). For the 8 mm threshold, the achieved sensitivity equaled 0.77, specificity was 0.66, and ACC reached 0.73.

Figure 4 illustrates the receiver operating characteristic (ROC) curve derived from the application of the 10 mm SAD threshold to our dataset. The ROC curve is a graphical plot that illustrates the diagnostic ability of a binary classifier system, as its discrimination threshold is varied. It is created by plotting the true positive rate (TPR, also known as sensitivity) against the false positive rate (FPR, or 1-specificity) at various threshold settings.

The red dot on the ROC curve represents the threshold point corresponding to the 10 mm cut-off for the short-axis measurement of the LN. It indicates the sensitivity and specificity balance achieved when applying the 10 mm SAD threshold criterion of 10 mm as the determinant factor for cancer metastasis classification. The area under the curve (AUC) for the 10 mm SAD threshold approach, denoted as AUC (10 mm SAD threshold), is 0.81, indicating a good diagnostic ability. Moreover, the accuracy (ACC (10 mm SAD threshold)) of the method is 0.71, which further substantiates the efficacy of the 10 mm SAD threshold in classifying tumor samples within this study’s framework. This is compared with the 8 mm threshold (green dot), which achieves sensitivity of 0.77, specificity equal to 0.66, and ACC reaching 0.73.

#### 2.3.2. Software

The overall data cleaning, analysis, as well as ML model preparation and processing were performed with the Python programming language (version 3.12.3) [57], Scikit-learn(version 0.8) [55] library and PyTorch framework (version 2.2) [58]. The Python language is a vastly used one when applications such as data cleaning, analysis, and visualization are concerned, with extensive libraries both for those approaches as well as various AI-focused solutions. One such library, both open source and commercialy usable, is Scikit-Learn. It provides tools for data preprocessing, predictive data analysis, classification, regression, clustering, dimensionality reduction, model selection, and other related tasks, making it a good start for any ML application. Finally, PyTorch is a framework for building deep learning models. It is prepared in Python and incorporates the use of a GPU into the computations.

#### 2.3.3. Multinomial Logistic Regression (MLR)

In the case of the LN-RADS scale, one serious problem is that the final outcome of the classification procedure refers to more than two labels. In this case, instead of two class definitions, as is the case with standard, histopathological data, the final outcome falls into a total of six possible classes in the LN-RADS scale. In that aspect, an additional algorithm is required. For this purpose, a Multinomial Logistic Regression algorithm was chosen [59]. This approach to the classification extends the logistic regression method to the problem with more than two classes.

#### 2.3.4. Training Using Histopatological Data

**Figure 5:**
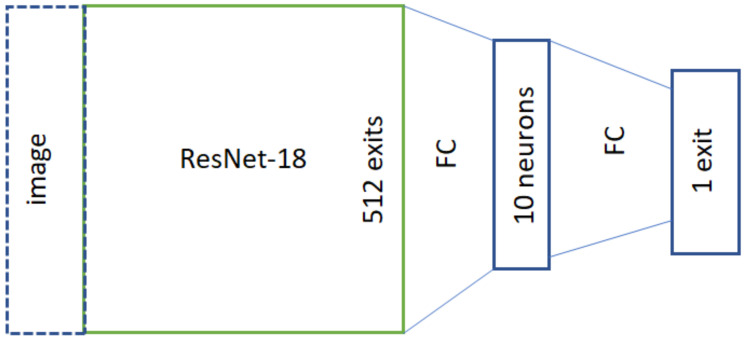
Network structure for the training process using histopathological data.

The first considered approach used histopathological data for the training process. The samples were divided according to expert evaluation, where each of them either contained or did not contain a cancerous LN. The original network structure is presented in Figure 5. In this approach, only a binary variable (cancer/no cancer) is used for the training process, and the network itself has a narrower architecture (denoted as 1a_hist_narrow).

**Figure 6:**
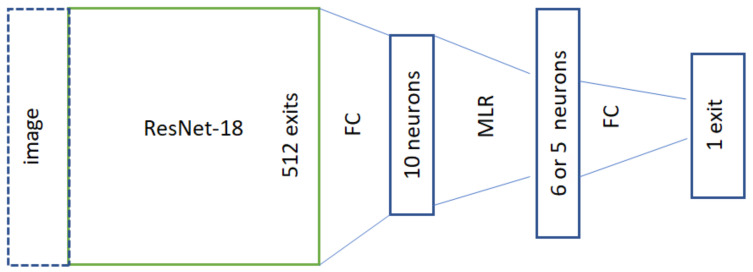
Network structure for the training process using histopathological data, with additional MLR layer.

To achieve better comparability with the LN-RADS scale approach, an alternative structure was used. In this case, the network was extended with an additional 5 or 6 neurons before the last network layer, with the MLR approach between the original 10 neurons obtained after transferring the image through ResNet18 network and the final output (denoted as 1b_hist_wide). The network architecture, after this modification, is presented in Figure 6.

#### 2.3.5. Training Using LN-RADS Scale

**Figure 7:**
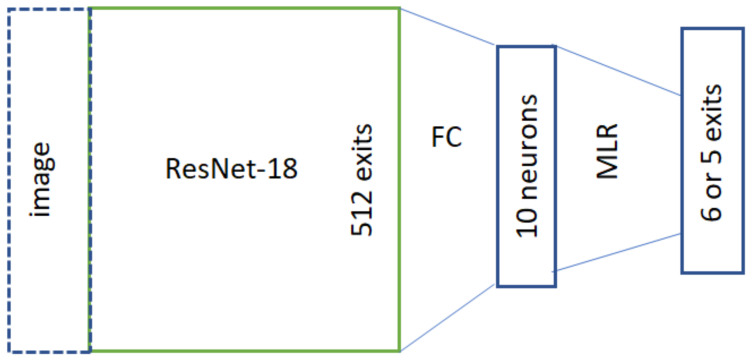
Network structure used for the training using data denoted with the LN-RADS scale.

The second approach uses the LN-RADS scale for the training process. Since in this case there are total of six possible labels for any data, the MLR algorithm is used for classification. In order to describe the variable itself, it was coded according to values outlined in Table 4. The original version of this approach uses codes with an additional column of ones. It was noted that this can be omitted, reducing the code length to five values. For example, using this coding, the label 4a will be denoted as [1, 1, 1, 0, 0], while in the original version, it would be described with six values as [1, 1, 1, 1, 0, 0] with an additional ‘1’ in the first place. Depending on the chosen representation, this network layer can have either six (original code table) or five exits (in the case of the approach with the reduced first column used in this paper). This approach was chosen since it includes the information about class order, which can also relate to the probability of the sample containing cancerous LNs and should be retained. The network structure used for training is presented in Figure 7.

In the case of the training using the LN-RADS scale, two additional approaches were used for the network layer marked as MLR. The first approach used a fully connected layer in this place instead of the classical MLR approach. The second approach used the actual Multinomial Logistic Regression structure, with five neuron exits. The absolute term is calculated separately for each of them, while the remaining parameters stay the same.

In general, due to the broadcasting used, each *i*-th bit (form 5 or 6, depending on the type of codingused) will correspond directly to the *i*-th output:

(1) $$y_{i}=sigmoid\left(a_{1}x_{1}+a_{2}x_{2}+\cdots +a_{10}x_{10}+b_{i}\right)$$

In this case, the parameters $a_{1},a_{2},\cdots ,a_{10}$ are trained similarly as other network values while remaining the same at the level of each bit (*i*-th output). The only parameter dependent on the *i* value is the constant index “b” or bias. The presented network generates a five-element vector with values in the range 0–1. This form allows the output to be tied more closely to the monotonicity of the network response. The score can be calculated as follows:

(2) $$\frac{1}{5}\sum_{i=1}^{5}y_{i}$$

which is monotonous in relation to $a_{1}x_{1}+a_{2}x_{2}+\cdots +a_{10}x_{10}$.

Having true answers as to whether cancer was found in the node (original, histopathological data with expert denotation), the AUC can be calculated. When it comes to ACC or measures based on the confusion matrix, thresholding is necessary. Here, the score was compared against the value that maximizes the true positive rate with the false positive rate (based on the training set). This method was denoted as: if_pure_mulitinomial=True.

Regardless of the approach used, the average of all five outputs is taken for the thresholding, while the actual threshold is calculated from the test set containing histopathological data.

## 3. Results

Apart from prediction accuracy, two quality measures are used in order to evaluate the results.The first one is the ROC measure. It was chosen since it shows the performance of the given classification at all thresholds. The ROC curve plots two parameters: the true positive rate and the false positive rate. Additionally, in the case of LN classification using the 10 mm SAD threshold, it was decided that this will show the most relevant information.

The second important parameter is the AUC (area under ROC curve). It provides an aggregated performance measure for all classification thresholds since it measures the entire two-dimensional area under the ROC curve. In general, it can be interpreted as a probability that the model will rank a random positive example higher than random negative one. This measure is scale-invariant (measuring how well predictions are made, rather than focusing on their values) and classification-threshold-invariant (checking prediction quality irrespective of the chosen classification threshold). Both measures are important in the evaluation of the achieved solutions.

During experiments, the first tested element was the baseline performance achieved by using the 10 mm SAD threshold. In this case, no learning happened, and the samples were classified according to the given descriptions. Figure 4 shows the ROC curve (receiver operating characteristic curve) for this classification. For the threshold classification point of 10 mm (measuring the short axis in the cross section of the LN), the classification accuracy was equal to 0.72, with the AUC parameter equal to 0.81. Additionally, for this point there were a total of 147 true-negative, 138 true-positive, 88 false-negative, and 25 false-positive examples. Those values were later used as a baseline for the AUC and ACC (accuracy) scores achieved by the remaining solutions.

We analyzed the performance of several diagnostic methods using two main metrics: accuracy (ACC) and area under the receiver operating characteristic curve (AUC). The methods were tested against a baseline represented by the 10 mm SAD threshold. The following subsections detail the performance trends observed across 100 epochs for each method.

### 3.1. Accuracy (ACC) Performance

**Figure 8:**
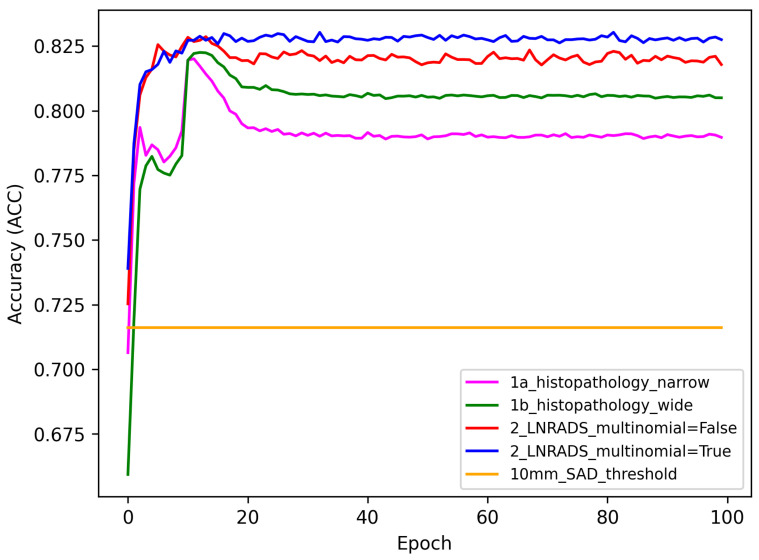
Accuracy results for the given method set with the 10 mm SAD threshold used as a baseline for testing.

The accuracy results, presented in Figure 8, show how each method’s ability to correctly diagnose cases evolves over time. The baseline 10 mm SAD threshold accuracy is depicted by an orange line and remains constant at approximately 0.7.

The ‘1a_histopathology_narrow’ method, visualized by the purple line, and the ‘1b_histopathology_wide’ method, in green, both exhibit an initial increase in accuracy, with the narrow criteria peaking slightly earlier than the wide one. After the initial increase, both methods plateau, indicating a period of stability in diagnostic accuracy with minimal improvement.

For the ‘2_LN-RADS_multinomial=False’ method (red line), there is a rapid climb to peak accuracy, surpassing both histopathological methods. The ‘2_LN-RADS_multinomial=

True’ method (blue line) closely follows the trend of the red line, suggesting that the use of multinomial regression in LN-RADS methods does not significantly alter the accuracy trajectory in comparison to the non-multinomial model.

### 3.2. AUC Performance

**Figure 9:**
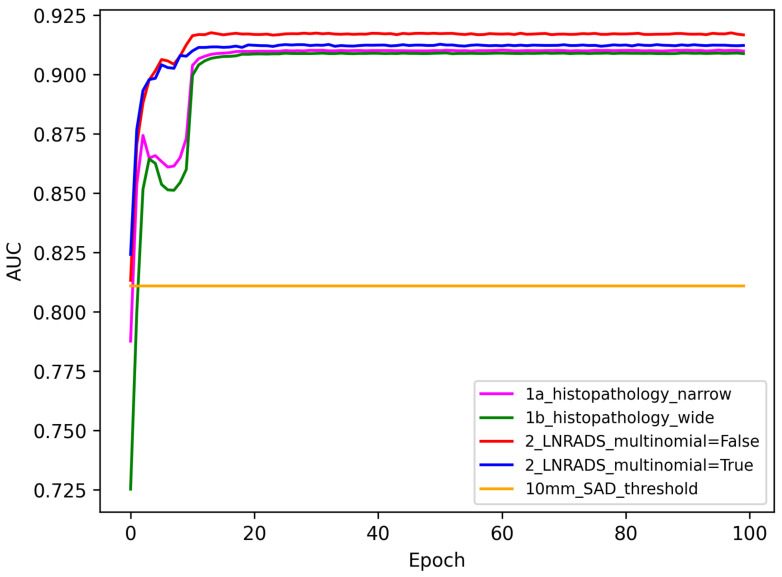
AUC results for the given method set with the 10 mm SAD threshold used as a baseline for testing.

As illustrated in Figure 9, the AUC metric provides insights into the probability that the models will rank a randomly chosen positive instance higher than a negative one. The 10 mm SAD threshold baseline is constant at an AUC of about 0.8.

All methods quickly exceed the 0 mm SAD threshold AUC baseline, with the ‘1a_histo pathology_na-rrow‘ and ‘1b_histopathology_wide‘ methods converging to a similar AUC after initial fluctuations. Both LN-RADS methods (red and blue lines) demonstrate superior performance over the histopathological methods, maintaining an AUC close to 0.9 after the initial learning period.

The comparison between the accuracy and AUC results suggests that while the LN-RADS methods consistently offer higher performance metrics, the choice of multinomial regression does not seem to influence the outcomes significantly. Both histopathology and LN-RADS methods show promise over the 10 mm SAD threshold baseline, with the LN-RADS methods appearing to be more robust.

### 3.3. Comparative Analysis of Model Performance

Table 5 presents the performance metrics for various diagnostic models evaluated in the study. The models include both histopathology-based approaches and LN-RADS-based methods, along with a traditional 10 mm SAD threshold for comparison. The metrics considered for evaluation are accuracy, specificity, sensitivity, precision, and F1 score. These metrics provide a comprehensive understanding of each model’s diagnostic capabilities and limitations.

- 1a Histopathology Narrow: This model exhibits a balanced performance with a high specificity of 93.08% and a reasonable accuracy of 78.97%. Its sensitivity and precision are 68.23% and 92.83%, respectively, with an overall F1 score of 78.65%.
- 1b Histopathology Wide: Demonstrating an improvement over the narrow model, this approach achieves an accuracy of 80.50%. It shows a slightly lower specificity of 87.37% but a better sensitivity of 75.27%. The precision and F1 score are 88.68% and 81.42%, respectively.
- 2 LNRADS Cut (Multinomial = False): This model, using the LN-RADS scale without multinomial regression, scores an accuracy of 81.78%. Its specificity and sensitivity are 88.90% and 76.37%, respectively, while maintaining a precision of 90.04%. The F1 score is 82.64%, indicating a robust balance between precision and recall.
- 2 LNRADS Cut (Multinomial = True): Incorporating multinomial regression into the LN-RADS based model, it slightly improves the accuracy to 82.75%. The specificity slightly decreases to 88.47% compared to its counterpart, but it records a higher sensitivity of 78.39%. Precision and F1 score are 89.93% and 83.77%, respectively.
- 10 mm SAD Threshold: Serving as the baseline, this traditional method shows the lowest performance, with an accuracy of 71.61%. Its specificity is 85.47%, and sensitivity is notably lower at 61.06%. Precision and F1 score are 84.66% and 70.95%, respectively.

The above results highlight the enhanced effectiveness of AI-based models over the conventional 10 mm SAD threshold method, particularly in terms of accuracy and sensitivity. Both histopathology- and LN-RADS-based approaches demonstrate promising capabilities, with the latter showing a slight edge in overall performance.

## 4. Discussion

The main goal of the research presented in this paper was to show that the proposed AI-based solutions for LN cancer diagnosis perform better on LN-RADS than on histopatology data. Also, it is better than the approach based solely on the 10 mm SAD threshold. As clearly shown by the results, all proposed methods achieved higher ACC and AUC scores, outperforming the baseline solution by a large margin.

The problem of identifying LNs in terms of detecting malignant lesions is one of the key issues that determines the course of further treatment and the prognosis. Until recently, it was believed that the key parameter was the diameter of the node in the short axis. It turns out, however, that the mentioned value is not a parameter that determines the nature of changes in a given node.

To properly assess the condition (and therefore the risk of cancer), it is crucial to take a closer look at the appearance/structure of a given node. The ability to do so early is crucial to the continued success of treatment. This is, among other things, the reason for the interest in artificial intelligence methods in the above-mentioned diagnostic process. If supervised learning models are used, one of the key questions includes target type—or what element should be presented for the network as the desired answer. While histopathological labels indicating samples as cancerous or not cancerous can be a solution, it turns out it might not be the best approach, especially with USG images in their original form. This can lead to distortions since a lot of samples will not contain cancerous cells, for example, due to the fact that they were destroyed during the treatment. In this case the sample will look like cancer, without containing the actual cells, negatively influencing network accuracy.

The malignancy predictor—SAD—depending on the cut-off point value, has higher sensitivity or specificity; in the case of a value of 10 mm, it does not detect approximately 22% of metastatic nodes compared to the LN-RADS criteria. This is because SAD is not the best predictor of cancer, as many authors point out. They note, which is consistent with our results, that changes in lymph nodes show some differences in the case of inflammatory and neoplastic processes.

In [60], it is pointed out that in newly diagnosed breast cancer patients, increasing axillary LN cortical thickness, abnormal fatty hilum, and diffuse cortical thickening are associated with nodal metastasis. PPVs of axillary LN cortical thickness ≥ 3 mm and ≥3.5 mm are similar but increase for cortical thickness ≥ 4 mm. FNA of axillary LNs with cortex < 4 mm may be unnecessary for some patients undergoing sentinel LN biopsy. In [61], the authors point out that in the case of an inflammatory or post-vaccination reaction, the changes are more diffuse, while in the case of cancer, the predictor is an increase in the thickness of the cortex, especially local or focal with local bulging of the cortex and modeling or displacement.

In cases of inflammation, the process spreads more easily to the entire node and the cortex of the node thickens more evenly (diffuse), while in the case of tumor cells, there is a tendency for the focal growth of tumor cell colonies forming more local cortical thickening (LCT) or focal (focal cortical thickenning—FCT) thickening of the cortex.

The obtained results confirm general assumptions in that aspect. The model trained on histopathological data still performs better than the baseline. The wider network structure achieved higher ACC results, while both of them have very similar AUC scores, with slight superiority of the narrower network. At the same time, both approaches using the LN-RADS scale achieved higher results in terms of AUC and ACC values than solutions based on histopathological data. It can be seen that from the point of view of the quality of the network in terms of discriminating cancer/non-cancer lesions, information about the LN-RADS scale is more valuable for the training. When it comes to comparison between both top performing approaches, the method using the MLR in the network structure achieves a higher ACC score, while the solution containing a layer with fully connected neurons in its place reaches a higher AUC value.

While it is clear that the proposed solutions are very promising, especially in terms of achieved scores and the possibility to automate the diagnostic process, there are still quite a few areas of further research that can be addressed. Any insight gained during such experiments can lead to possible improvements in the final solution and overall diagnosis accuracy. Secondly, since the network structure impacts achieved scores, testing and evaluating different designs might bring some additional information. Another possible improvement is to combine both datasets and prepare a solution trained on the LN-RADS scale with histopathological labels included, expanding the data used during the decision process. Preparing and evaluating such a solution can bring additional insights and possibly further raise achieved results.

The performance of various diagnostic models was evaluated based on metrics such as accuracy, specificity, sensitivity, precision, and F1 score. Each model’s performance sheds light on its effectiveness in diagnosing cancerous LNs.

Histopathology Models: Both the 1a_histopathology_narrow and 1b_histopathology

_wide models demonstrated significant improvements over the 10 mm SAD threshold baseline. The wider network structure of the 1b model showed better accuracy, suggesting that a more comprehensive network can capture the nuances of histopathological data more effectively.

LN-RADS Models: The LN-RADS-based models, both with and without multinomial regression, outperformed the histopathology models in terms of accuracy and AUC. This indicates that the LN-RADS scale provides valuable information for AI models to differentiate between cancerous and non-cancerous LNs more effectively. The slightly higher performance of the model incorporating multinomial regression suggests that this approach may capture the ordinal nature of the LN-RADS scale more effectively.

Baseline Model (10 mm SAD Threshold): As expected, the traditional 10 mm SAD threshold method showed the lowest performance among all tested models. This highlights the limitations of relying solely on size measurements for diagnosing LN cancer and underscores the need for more sophisticated diagnostic methods.

Overall Insights: The results indicate that AI-based models, particularly those utilizing the LN-RADS scale, can significantly improve the accuracy of LN cancer diagnosis. These models not only provide more accurate diagnoses but also offer a deeper understanding of the disease’s progression, which is critical for effective treatment planning.

Future research should focus on further refining these models, exploring the integration of additional diagnostic parameters and validating the results in a clinical setting.

Overall, the main hypothesis of this paper was confirmed since all of the evaluated approaches were able to significantly improve the score achieved by using the 10 mm SAD threshold. It can be clearly seen that solutions based on artificial intelligence can improve the diagnostic accuracy as well as speed up the process, possibly improving patient prognosis by identifying cancerous LNs at an earlier development stages.

## 5. Conclusions

The findings of this study highlight the potential of AI-assisted ultrasound imaging and the LN-RADS scale in improving the detection of metastatic lymph nodes. In general, the performed experiments show that AI-based solutions for LN cancer diagnosis perform better on the LN-RADS scale than on histopatology data. However, it is essential to acknowledge the limitations and address potential confounding factors that may impact the interpretability and generalizability of the results.

One of the key challenges encountered in this research was the limited sample size, which could potentially introduce bias and restrict the generalizability of the findings. While the dataset comprised 398 ultrasound images, a larger and more diverse sample would enhance the robustness of the analysis and increase confidence in the applicability of the proposed methods across different patient populations and clinical settings.

Moreover, the retrospective nature of the study design may introduce inherent biases related to data collection and analysis. Prospective studies with well-defined inclusion and exclusion criteria, as well as rigorous data collection protocols, would further validate the conclusions drawn from the presented research.

Another aspect that warrants further investigation is the potential impact of inter-observer variability on the LN-RADS assessments. Despite the positive inter-reader agreement, it is crucial to evaluate the consistency and reproducibility of LN-RADS classifications across a broader range of radiologists and clinical settings. Standardized training and quality control measures may be required to ensure consistent application of the LN-RADS criteria and minimize potential biases.

Overall, the main hypothesis of the paper was confirmed, with all AI-based approaches achieving better results than the baseline solution using the 10 mm SAD threshold. The findings of the study require further investigation and evaluation but are promising in the aspect of potential improvements to the accuracy, speed, and overall LS node classification.

## Figures and Tables

**Table 1:** Distribution of lymph node samples by location.

No.	Location	Number of Cases	Percentage [%]
1	Axilla	153	38.44
2	Neck	148	37.19
3	Groin	79	19.85
4	Other	18	4.52
-	**Total**	398	100

**Table 2:** Dataset structure for samples classified using LN-RADS scale.

LN-RADS Label	Cancer/No Cancer	Number of Cases	Percentage [%]
1	NO	21	5.28
2	NO	28	7.04
3	NO	57	14.30
3	YES	1	0.25
4a	NO	53	13.32
4a	YES	30	7.54
4b	NO	12	3.02
4b	YES	77	19.35
5	NO	1	0.25
5	YES	118	29.65
**Total**	172/226	398	100

**Table 3:** Distribution of cancer types based on histopathology results.

No.	Type of Cancer	Number of Cases	Percentage [%]
1	Breast Cancer	78	34.51
2	Lymphoma and Leukemia	38	16.81
3	Melanoma	24	10.62
4	FPI—origin unknown	23	10.18
5	Head and Neck Cancer	22	9.73
6	Other	17	7.52
7	Colorectal Carcinoma	13	5.75
8	Lung Cancer	11	4.87
-	**Total**	226	100

**Table 4:** LN-RADS scale labels with associated code.

LN-RADS			Code		
1	0	0	0	0	0
2	1	0	0	0	0
3	1	1	0	0	0
4a	1	1	1	0	0
4b	1	1	1	1	0
5	1	1	1	1	1

**Table 5:** Performance metric comparison of diagnostic models.

Model	Accuracy	Specificity	Sensitivity	Precision	F1 Score
1a Histopathology Narrow	78.97%	93.08%	68.23%	92.83%	78.65%
1b Histopathology Wide	80.50%	87.37%	75.27%	88.68%	81.42%
2 LNRADS Cut (Multinomial = False)	81.78%	88.90%	76.37%	90.04%	82.64%
2 LNRADS Cut (Multinomial = True)	82.75%	88.47%	78.39%	89.93%	83.77%
10 mm SAD threshold	71.61%	85.47%	61.06%	84.66%	70.95%

## Data Availability

Data available on request due to restrictions. The data presented in this study are available on request from the corresponding author. The data are not publicly available due to the medical nature of original images.
